# Supplementary material for: Anomalous Hall effect from inter-superlattice scattering in a noncollinear antiferromagnet
Source: Nat Commun. 2025 Jul 1;16:5711. doi: 10.1038/s41467-025-61211-4 (PMC12216071; doi:10.1038/s41467-025-61211-4)
Supplement: Supplementary file 1 — Supplementary Information [file 41467_2025_61211_MOESM1_ESM.pdf]

Supplementary Information: Anomalous Hall effect from inter-superlattice scattering in a noncollinear antiferromagnet

Lilia S. Xie[1,*,+], Shannon S. Fender[1], Cameron Mollazadeh[1], Wuzhang Fang[2], Matthias D. Frontzek[3], Samra Husremović[1], Kejun Li[2,4], Isaac M. Craig[1], Berit H. Goodge[1,5], Matthew P. Erodici[1], Oscar Gonzalez[1], Jonathan D. Denlinger[6], Yuan Ping[2], and D. Kwabena Bediako[1,7,8,*]

[1]*Department of Chemistry, University of California, Berkeley, CA 94720, USA*

[2]*Department of Materials Science and Engineering, University of Wisconsin, Madison, WI, 53706, USA*

[3]*Neutron Scattering Division, Oak Ridge National Laboratory (ORNL), Oak Ridge, Tennessee 37831, USA*

[4]*Department of Physics, University of California, Santa Cruz, CA, 95064, USA*

[5]*Max-Planck-Institute for Chemical Physics of Solids, Nöthnitzer Str. 40, 01187, Dresden, Germany*

[6]*Advanced Light Source, Lawrence Berkeley National Laboratory, Berkeley, CA 94720, USA*

[7]*Chemical Sciences Division, Lawrence Berkeley National Laboratory, Berkeley, CA 94720, USA*

[8]*Kavli Energy NanoScience Institute, Berkeley, CA 94720, USA*

[*]*Correspondence to: liliaxie@princeton.edu and bediako@berkeley.edu*

[+]*Present address: Department of Chemistry and the Princeton Materials Institute, Princeton University, Princeton, NJ 08544, USA*

Contents

1 Single-Crystal X-ray Diffraction **S3**

2 Energy-Dispersive X-ray Spectroscopy **S6**

3 Single-Crystal Neutron Diffraction **S7**

4 Anomalous Hall Conductivity **S9**

5 Magnetometry **S11**

6 Angle-Resolved Photoemission Spectroscopy **S19**

7 Density Functional Theory Calculations: Band Structure **S20**

8 Magnetotransport of $Cr_{1/4}TaS_2$ **S22**

9 Characterization of $Cr_{0.23}TaS_2$ **S23**

1 Single-Crystal X-ray Diffraction

Table S1: Crystal data and refinement details for $Cr_{1/4}TaS_2$ from single-crystal X-ray diffraction.

Empirical formula	$CrTa_4S_8$
Formula weight (g/mol)	1032.27
Temperature (K)	293(2)
Wavelength (Å)	0.71073
Crystal system	Hexagonal
Space group	$P6_3/mmc$
a (Å)	6.5959(3)
c (Å)	12.0391(7)
Volume (Å^{-3})	453.60(5)
Z	2
Density (calculated) (g/cm^3)	7.558
Absorption coefficient (mm^{-1})	50.985
$F(000)$	888
Crystal size (mm^3)	$0.031 \times 0.023 \times 0.015$
θ (°)	3.384 to 29.622
Index ranges	$-9 \leq h \leq 8$
	$-8 \leq k \leq 9$
	$-16 \leq l \leq 16$
Reflections collected	18880
Independent reflections	272
Completeness to θ_{full}	0.975
Absorption correction	Semi-empirical from equivalents
Refinement method	Full-matrix least-squares on F^2
Data / restraints / parameters	272 / 0 / 18
Goodness-of-fit on F^2	1.331
Final R indices $[I > 2\sigma(I)]$	$R_1 = 0.0404$, $wR_2 = 0.1430$
R indices (all data)	$R_1 = 0.0438$, $wR_2 = 0.1450$
Largest diff. peak and hole (e Å^{-3})	4.46 and -4.24

Table S2: Atomic coordinates, Wyckoff positions, and equivalent isotropic displacement parameters for $Cr_{1/4}TaS_2$ from single-crystal X-ray diffraction.

Atom Label	x	y	z	Site	U_{iso}
Cr1	0	0	1/2	$2a$	0.0121(13)
Ta2	0	0	1/4	$2b$	0.0064(5)
Ta3	0.49435(7)	0.50565(7)	1/4	$6h$	0.0063(4)
S4	0.1667(3)	0.3334(5)	0.3809(3)	$12k$	0.0074(8)
S5	2/3	1/3	0.1188(5)	$4f$	0.0076(11)

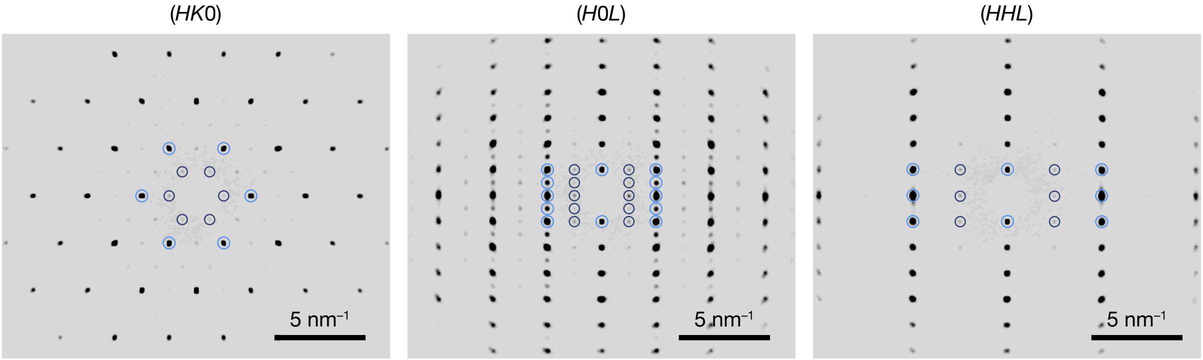

Figure S1: Reconstructed $(HK0)$, $(H0L)$, and (HHL) scattering planes from single-crystal X-ray diffraction of $Cr_{1/4}TaS_2$. Bragg peaks associated with the $2H$-TaS_2 host lattice and 2×2 Cr superlattice are circled in light blue and navy, respectively.

2 Energy-Dispersive X-ray Spectroscopy

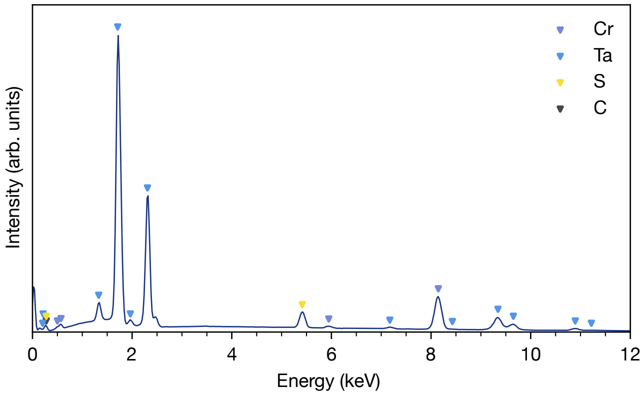

Figure S2: Representative energy dispersive X-ray spectroscopy data for a single crystal of $Cr_{1/4}TaS_2$, with peaks corresponding to Cr, Ta, S, and C labeled. The atomic ratio determined by fitting the Cr Kα_1, Ta Mα_1, and S Kα_1 peaks yields a formula of $Cr_{0.252(3)}TaS_{1.78(5)}$ for this crystal.

3 Single-Crystal Neutron Diffraction

Table S3: Refinement details for the nuclear structure of $Cr_{0.226(6)}TaS_2$ from single-crystal neutron diffraction.

Parameter	Value
Crystal mass (mg)	16.0
Temperature (K)	1.5
Wavelength (Å)	1.486
Space group	$P6_3/mmc$
a (Å)	6.619(5)
c (Å)	11.961(9)
Volume (Å^{-3})	453.8(6)
R_{F^2}	4.64
R_{wF^2}	6.36
R_F	5.05
$\chi^2(I)$	5.87
N_{eff} reflections	58 [$I > 2\sigma$]
Scale factor	693(12)

Table S4: Atomic coordinates, Wyckoff positions, isotropic displacement parameters, and occupancies for the nuclear structure of $Cr_{0.226(6)}TaS_2$ from single-crystal neutron diffraction.

Atom Label	x	y	z	Site	B_{iso}	Occupancy
Cr1	0	1	1/2	$2a$	1.73(7)	0.848(26)
Ta2	0	1	1/4	$2b$	1.73(7)	1
Ta3	0.495(1)	0.989(2)	1/4	$6h$	1.73(7)	1
S4	0.1670(9)	0.8330(9)	0.3802(5)	$12k$	1.73(7)	1
S5	$-1/3$	1/3	0.118(1)	$4f$	1.73(7)	1

Table S5: Refinement details for the magnetic structure of $Cr_{0.226(6)}TaS_2$ from single-crystal neutron diffraction.

Parameter	Value
Crystal mass (mg)	16.0
Temperature (K)	1.5
Wavelength (Å)	1.486
Representation	Γ_6
$\mathbf{k}_1$	(+1/3, +1/3, 0)
$\mathbf{k}_2$	(−1/3, −1/3, 0)
R_{F^2}	19.8
R_{wF^2}	30.8
R_F	10.2
$\chi^2(I)$	3.22
N_{eff} reflections	19 [$I > 2\sigma$]
Cr1 moment (μ_{B})	2.07(8)

4 Anomalous Hall Conductivity

The intrinsic anomalous Hall conductivity (AHC) can be calculated as:[1,2]

$$\sigma_{xy} = -\frac{e^2}{\hbar}\int_{\mathrm{BZ}} \frac{d\boldsymbol{k}}{(2\pi)^3}\Omega_{xy}(\boldsymbol{k}) \tag{S1}$$

where $\Omega_{xy}(\boldsymbol{k})$ is the total Berry curvature

$$\Omega_{xy}(\boldsymbol{k}) = \sum_n f_n(\boldsymbol{k})\Omega_{n,xy}(\boldsymbol{k}). \tag{S2}$$

n is the band index, and $f_n(\boldsymbol{k})$ is the occupation. $\Omega_{n,xy}(\boldsymbol{k})$ is the Berry curvature for the nth band which is calculated as

$$\Omega_{n,xy}(\boldsymbol{k}) = -2\hbar^2 \mathrm{Im} \sum_{m \neq n} \frac{\langle \psi_{n\boldsymbol{k}} | \hat{v}_x | \psi_{m\boldsymbol{k}} \rangle \langle \psi_{m\boldsymbol{k}} | \hat{v}_y | \psi_{n\boldsymbol{k}} \rangle}{[E_m(\boldsymbol{k}) - E_n(\boldsymbol{k})]^2}, \tag{S3}$$

where $\hat{v}$ is the velocity operator, ψ is the eigenstate, and E is energy. From the above equations, we see that AHC is determined by the total Berry curvature integrated over the Brillouin zone (BZ).

In magnetic materials, the Berry curvature and hence the intrinsic AHC is closely related to magnetic symmetry operations. Berry curvature behaves like an axial vector. If we apply a time-reversal operation, since the Berry curvature is odd with respect to a time-reversal operation $\mathcal{T}\Omega(\boldsymbol{k}) = -\Omega(-\boldsymbol{k})$, the Berry curvature will change the sign. Applying a time-reversal operation is equivalent to reversing the direction of magnetization of each sublattice. If the magnetic system has a symmetry such that $\mathcal{R}\Omega(\boldsymbol{k}) = -\Omega(\boldsymbol{k}')$, the Berry curvature is zero when integrating over the BZ, where $\mathcal{R}$ can be a mirror or rotation operation.[3] Such symmetry analysis has been applied to noncollinear antiferromagnet $GaNMn_3$ and explicitly verified by DFT calculations as reported in Ref. [4].

Magnetic ordering in $Cr_{1/4}TaS_2$ is determined by neutron scattering and shown in Figure S3. The associated magnetic space group is $P\overline{6}'2'm$, of which we consider two magnetic

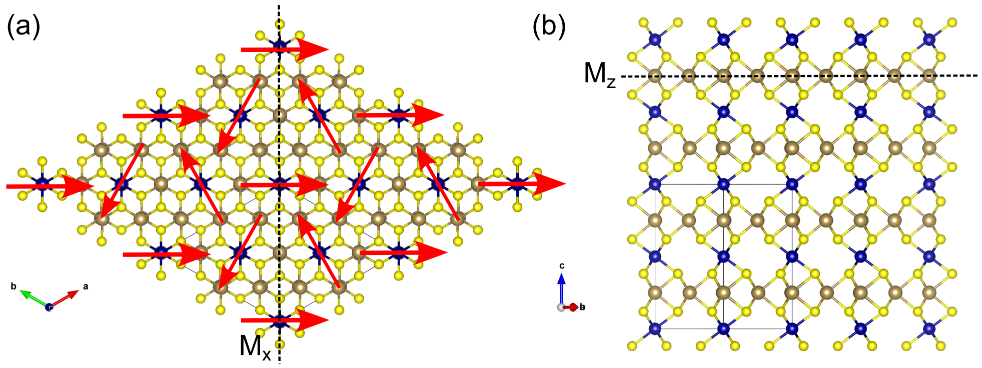

Figure S3: Magnetic ordering in $Cr_{1/4}TaS_2$ determined by neutron scattering. (a) top view (b) side view. Mirror operations $\mathcal{M}_x$ and $\mathcal{M}_z$ are labeled as dashed lines. Red arrows denote the magnetization of Cr atoms.

symmetry operations, $\mathcal{M}_x$ and $\mathcal{TM}_z$. Note that magnetization is also an axial vector, when applying a mirror operation, the component parallel to the mirror plane will reverse the direction, while the component perpendicular to the mirror plane will not. When applying these two symmetry operations to the Berry curvature, we have

$$\mathcal{M}_x\Omega^z(k_x, k_y, k_z) = -\Omega^z(-k_x, k_y, k_z) \tag{S4}$$

$$\mathcal{TM}_z\Omega^z(k_x, k_y, k_z) = \mathcal{T}\Omega^z(k_x, k_y, -k_z) = -\Omega^z(-k_x, -k_y, k_z). \tag{S5}$$

Either symmetry operation makes the Berry curvature an odd function in the $\boldsymbol{k}$ space, resulting in a zero Berry curvature when integrating the BZ. Our calculated AHC from DFT calculations is 1.6 S/cm, which is close to zero. We have also verified that the σ_{xy} component of AHC tensor in the magnetic space group $P\overline{6}'2'm$ is zero using the MTENSOR module at the Bilbao Crystallographic Server.[5]

The AHC is calculated using the OpenMX code[6,7] which utilizes pseudo-atomic orbitals as the basis functions.[8] Cr6.0-$s3p2d1$, Ta7.0-$s3p2d2f1$ and S7.0-$s2p2d1f1$ are specified as the basis functions. A cutoff energy of 300 Ry and a k-point grid of $4 \times 4 \times 4$ are used for self-consistent calculation with spin–orbit coupling. The AHC is calculated using a $24 \times 24 \times 24$ k-point grid.

5 Magnetometry

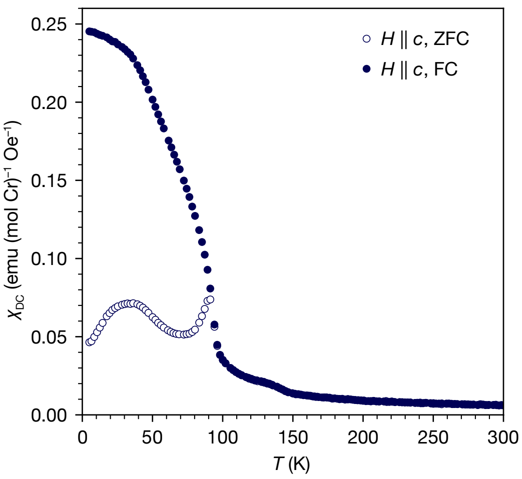

Figure S4: Field-cooled (FC) and zero-field-cooled (ZFC) DC magnetic susceptibility (χ_{DC}) vs. T, measured with a 100 Oe field perpendicular to c.

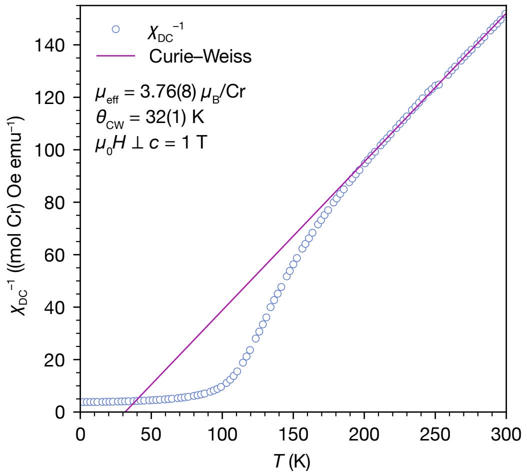

Figure S5: Fitting of inverse DC susceptibility data vs. T to the Curie–Weiss law, $\chi^{-1} = (T - \theta_{\text{CW}})/C$, for a 1 T magnetic field perpendicular to c.

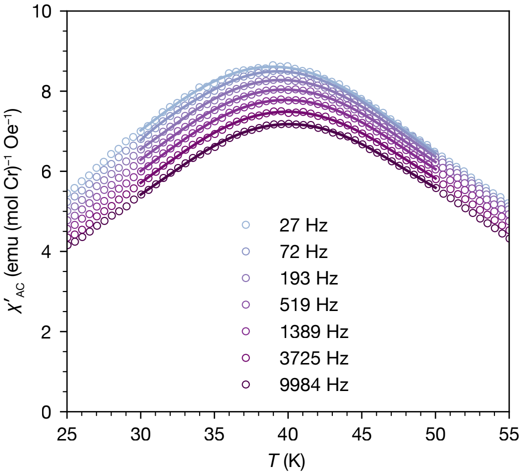

Figure S6: Real part of the AC susceptibility (χ'_{AC}), measured with an in-plane AC driving field of 10 Oe, fitted to a univariate spline in the vicinity of the spin glass transition temperature.

To extract the maximum of the real part of χ'_{AC}, corresponding to the spin glass freezing temperature (T_{f}) at each frequency, we fit the data to a univariate spline and take the root of the derivative. We then quantify the frequency dependence of T_f by calculating the Mydosh parameter (K), according to the formula $K = \frac{\Delta T_{\mathrm{f}}}{T_{\mathrm{f}} \log(\Delta f)}$ at different frequencies (f). We obtain $K = 0.011(4)$, which is somewhat larger than typical values for canonical spin glasses (e.g. 0.0045 for AuMn),[9] and in line with expected values for cluster spin glasses.[10–12]

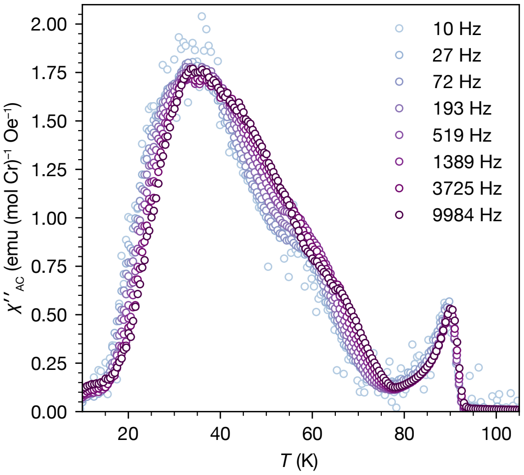

Figure S7: Imaginary part of the AC susceptibility (χ''_{AC}), measured with an in-plane AC driving field of 10 Oe.

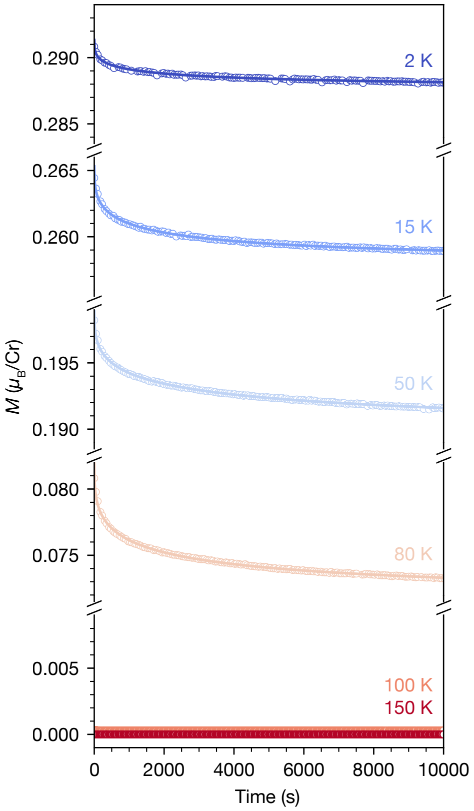

Figure S8: Thermoremanent magnetization curves obtained by field-cooling in a field of 1 T, removing the field, then measuring the magnetization as a function of time. Open circles represent the data, while solid lines represent fits to stretched exponential functions for 2 K, 15 K, 50 K, and 80 K.

Table S6: Parameters for fitting thermoremanent magnetization data to a stretched exponential function, $M(t) = M_0 + M_1 \exp\left[-(\frac{t}{\tau})^{1-n}\right]$, where $M(t)$ is the total magnetization, t is the time, M_0 is the constant magnetization, M_1 is the glassy component of the magnetization, τ is the characteristic relaxation time, and n is a stretching exponent.

T (K)	M_0 (μ_B/Cr)	M_1 (μ_B/Cr)	τ (s)	n
2	0.28768(2)	0.003641(6)	1224.4(4)	0.6541(7)
15	0.25815(2)	0.007156(6)	1231.3(2)	0.6274(4)
50	0.18978(5)	0.009451(9)	2315.6(5)	0.6586(4)
80	0.07196(3)	0.01006(7)	1425.8(2)	0.6399(3)

The obtained values for τ and n are consistent with those expected for a spin glass.[10] The persistence of thermoremanent magnetization above $T_f = 40$ K and below $T_C = 96$ K suggests that the FM state also exhibits some glassy characteristics.

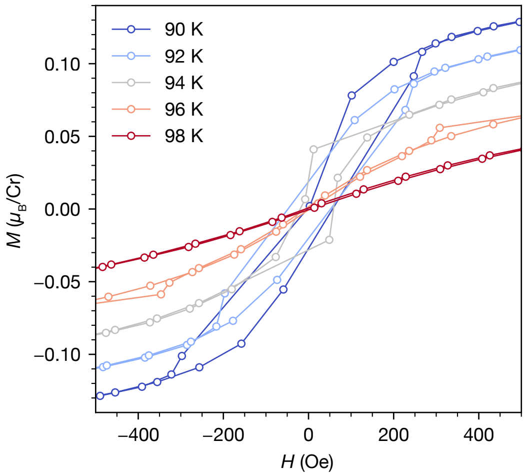

Figure S9: Isothermal magnetization curves with $H \perp c$, showing non-zero coercive field at 94 K and below.

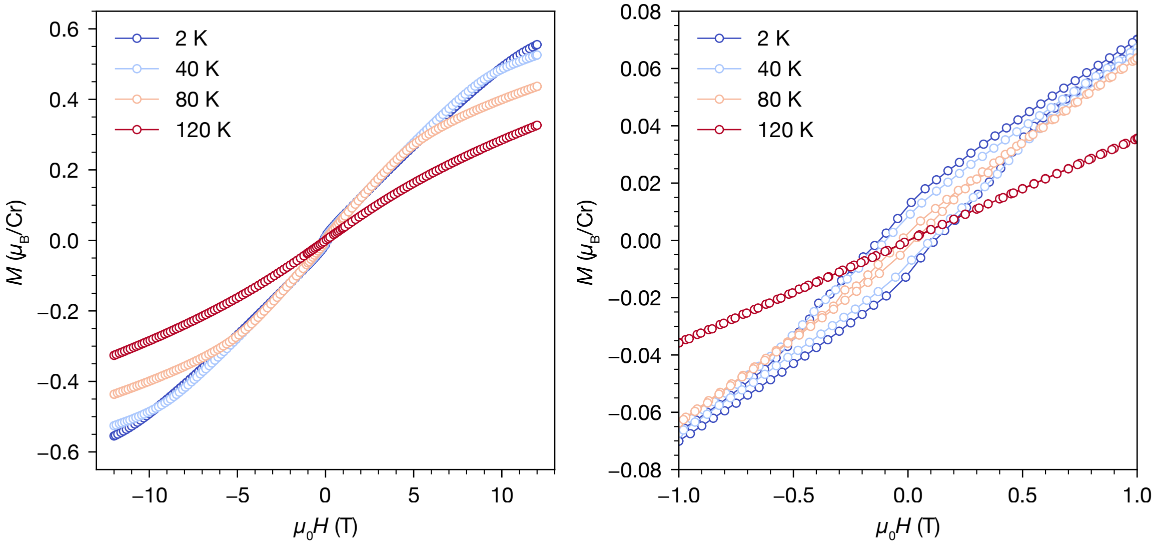

Figure S10: Isothermal magnetization curves with $H \parallel c$.

6 Angle-Resolved Photoemission Spectroscopy

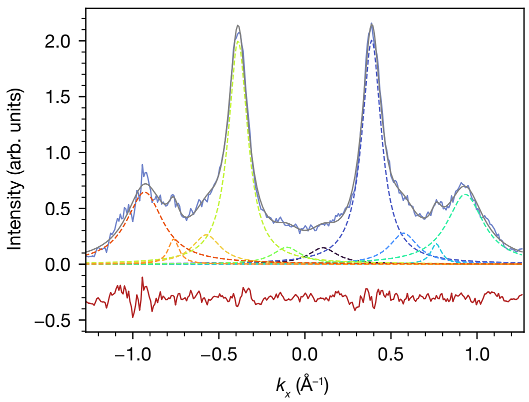

Figure S11: Momentum distribution curve for $E - E_F = 0$ eV and $k_y = 0$ Å^{-1}, fitted to multiple Lorentzian peaks.

7 Density Functional Theory Calculations: Band Structure

In Figure S12a–c, we show different unit cells of $2H$-TaS_2 from $1 \times 1 \times 1$, $2 \times 2 \times 1$, to $2\sqrt{3} \times 2\sqrt{3} \times 1$. One effect of increasing the unit cell size is the folding of the band structure in the reciprocal space, as seen in Figure S12d–f. In spite of the band folding, the calculated band structure of $2H$-TaS_2 always contains the key features that are measured from the ARPES experiment. The position of the key features in DFT calculation differs by 0.2 eV with respect to the experiment. The comparison indicates that the main contribution to ARPES measurement is from $2H$-TaS_2, and that the intercalated Cr shifts the Fermi level by introducing extra electrons.

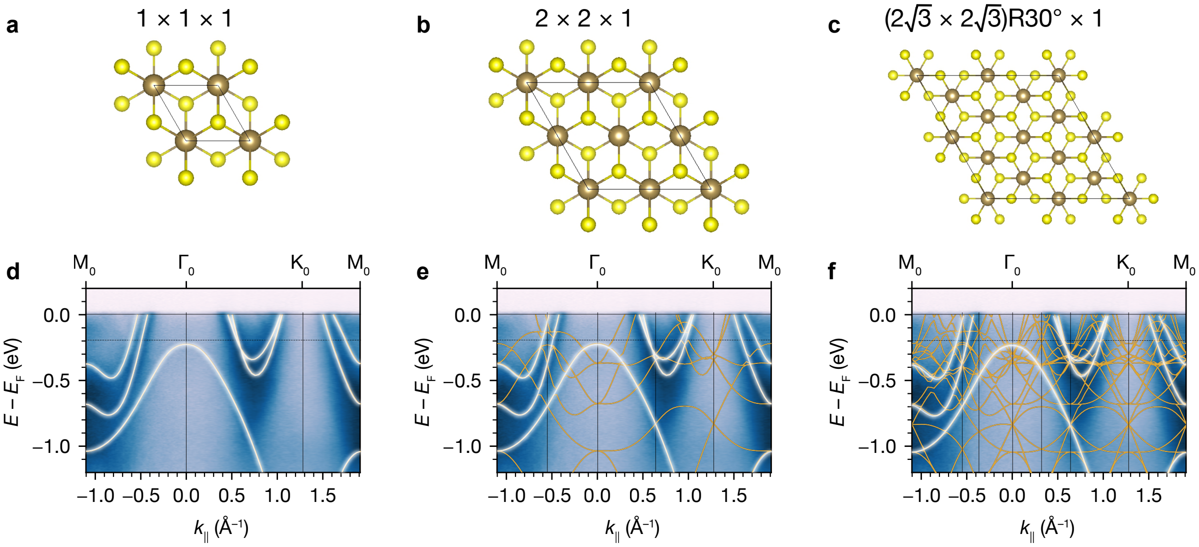

Figure S12: Increasing cell size and the folding of the bandstructure (yellow, shifted down by 0.2 eV) of $2H$-TaS_2 in the reciprocal space in comparison with the ARPES experiment. (a) $1 \times 1 \times 1$, (b) $2 \times 2 \times 1$, (c) $2\sqrt{3} \times 2\sqrt{3} \times 1$ unit cells of $2H$-TaS_2. The calculated bandstructure of (d) $1 \times 1 \times 1$, (e) $2 \times 2 \times 1$, (f) $2\sqrt{3} \times 2\sqrt{3} \times 1$ unit cells of $2H$-TaS_2.

In Figure S13a, we compare the band structures of $Cr_{1/4}TaS_2$ with $U = 0$ and $U = 4$ eV. Including Hubbard interaction moves the bands upward, which brings the agreement between DFT band structures and ARPES measurements closer. It also increases the magnetic moment on Cr from 2.5 μ_B to 3.2 μ_B, which makes it close to the theoretical spin-only value of 3.87 μ_B/Cr for Cr^{3+} ($S = 3/2$). In Figure S13b, we compare the band structures of $Cr_{1/4}TaS_2$ with different k_z. Increasing k_z makes many bands degenerate. Overall, including Hubbard

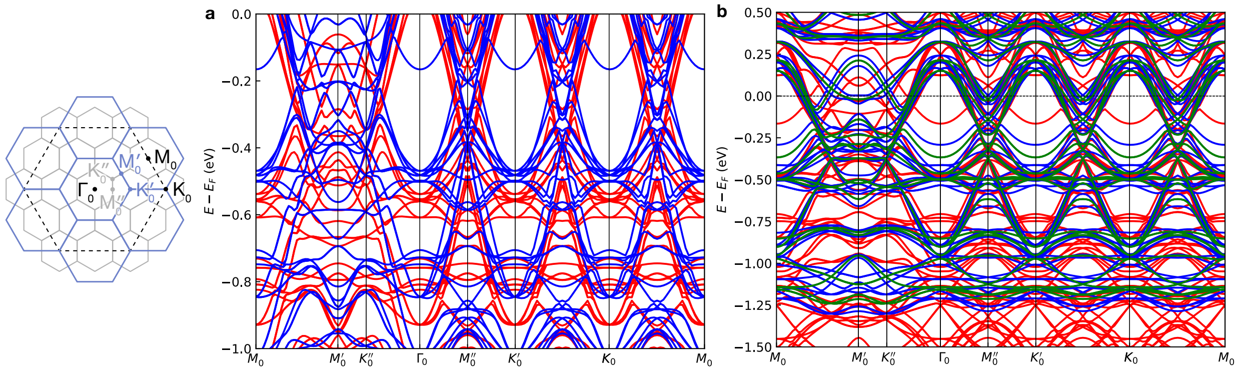

Figure S13: (a) Band structures of $Cr_{1/4}TaS_2$ with U=0 (red) and U=4 (blue) eV. (b) Band structures of $Cr_{1/4}TaS_2$ with $k_z = 0$ (red), $k_z = 0.4$ (blue), and $k_z = 0.5$ (green).

U with a $k_z = 0.5$ gives the best agreement between DFT band structures and the main ARPES dataset collected with $h\nu = 80$ eV.

8 Magnetotransport of $Cr_{1/4}TaS_2$

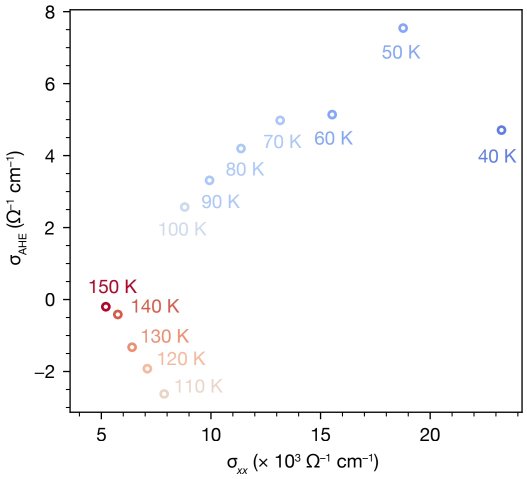

Figure S14: Plot of the longitudinal conductivity (σ_{xx}) vs. the anomalous Hall conductivity (σ_{AHE}).

9 Characterization of $Cr_{0.23}TaS_2$

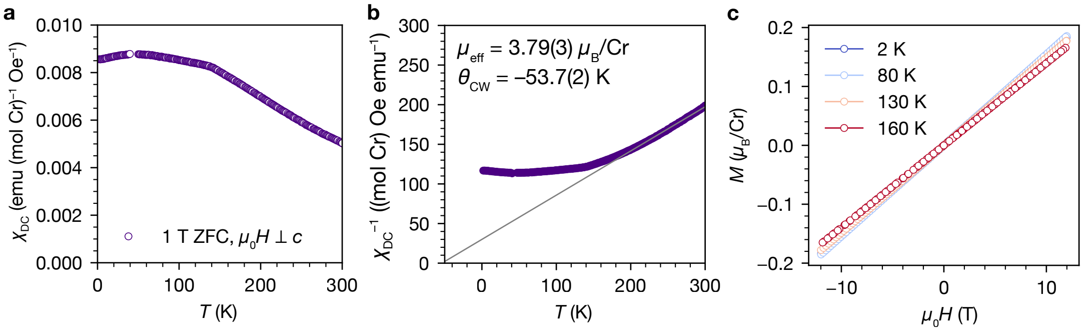

Figure S15: Magnetometry of $Cr_{0.23}TaS_2$. (a) Zero-field-cooled (ZFC) DC magnetic susceptibility (χ_{DC}) vs. T, measured with a 1 T magnetic field perpendicular to c. (b) Fitting of inverse DC susceptibility data vs. T to the Curie–Weiss law, $\chi^{-1} = (T - \theta_{CW})/C$. (c) Isothermal magnetization (M) vs. $\mu_0 H \perp c$.

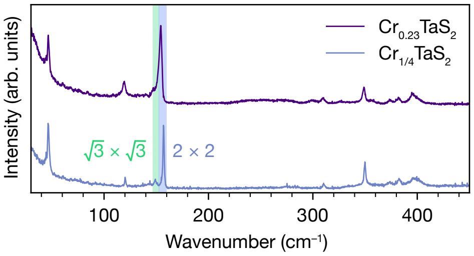

Figure S16: Raman spectra of $Cr_{0.23}TaS_2$ and $Cr_{1/4}TaS_2$, with $\sqrt{3}\times\sqrt{3}$ and 2×2 superlattice peak regions highlighted for $Cr_{1/4}TaS_2$.

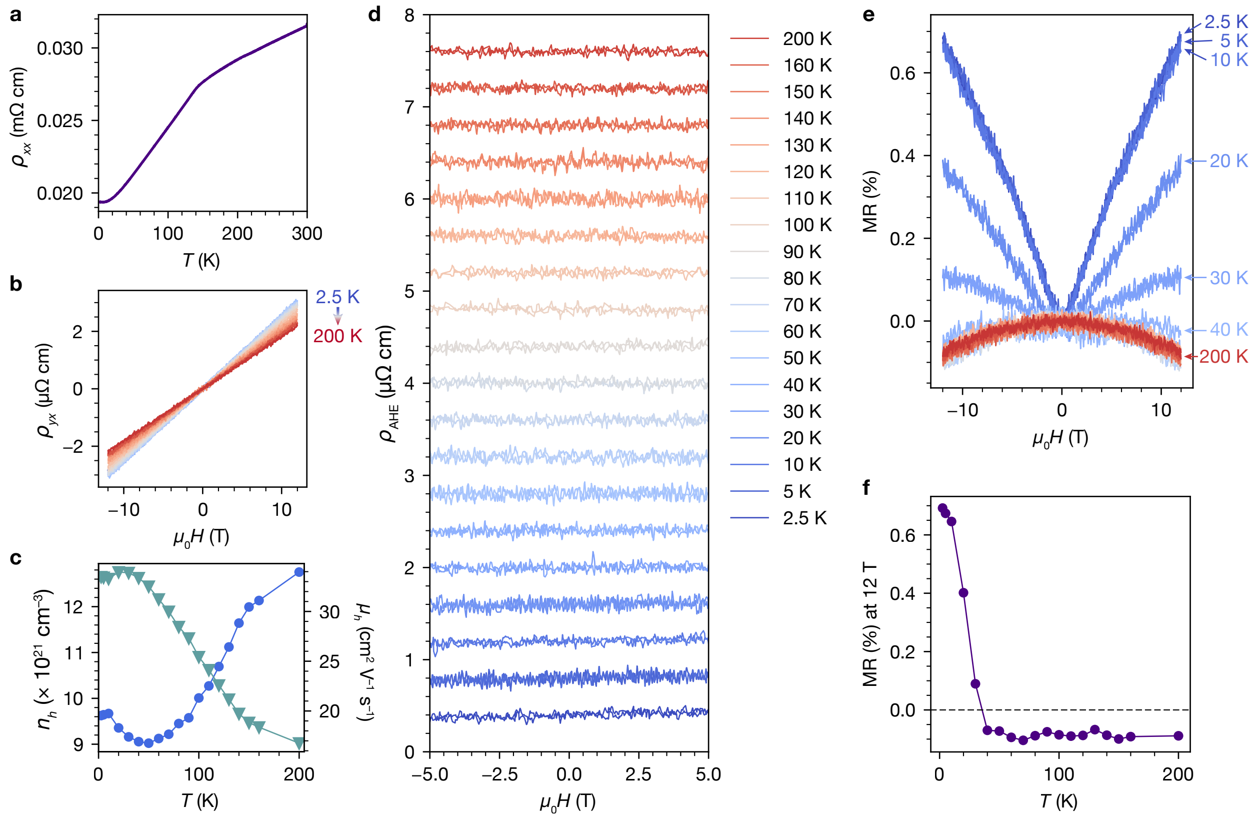

Figure S17: Magnetotransport properties of $Cr_{0.23}TaS_2$. (a) Longitudinal resistivity (ρ_{xx}) vs. T. (b) Hall resistivity (ρ_{yx}) vs. $\mu_0 H$. (c) Charge carrier concentration (n_h) and carrier mobility (μ_h) vs. T, as derived from the ordinary Hall component of ρ_{yx}. (d) Anomalous Hall resistivity (ρ_{AHE}) vs. $\mu_0 H$ at different temperatures, obtained by subtracting the ordinary Hall component of ρ_{yx}. (e) Magnetoresistance (MR) vs. $\mu_0 H$ at different temperatures. (f) MR at 12 T vs. T.

Supplementary References

1. Wang, X., Yates, J. R., Souza, I. & Vanderbilt, D. Ab initio calculation of the anomalous Hall conductivity by Wannier interpolation. *Phys. Rev. B* **74,** 195118 (2006).
2. Nagaosa, N., Sinova, J., Onoda, S., MacDonald, A. H. & Ong, N. P. Anomalous Hall effect. *Rev. Mod. Phys.* **82,** 1539–1592 (2010).
3. Suzuki, M.-T., Koretsune, T., Ochi, M. & Arita, R. Cluster multipole theory for anomalous Hall effect in antiferromagnets. *Phys. Rev. B* **95,** 094406 (2017).
4. Gurung, G., Shao, D.-F., Paudel, T. R. & Tsymbal, E. Y. Anomalous Hall conductivity of noncollinear magnetic antiperovskites. *Phys. Rev. Mater.* **3,** 044409 (2019).
5. Gallego, S. V., Etxebarria, J., Elcoro, L., Tasci, E. S. & Perez-Mato, J. M. Automatic calculation of symmetry-adapted tensors in magnetic and non-magnetic materials: a new tool of the Bilbao Crystallographic Server. *Acta Cryst. A* **75,** 438–447 (2019).
6. T. Ozaki *et al.*, OpenMX 3.9. `http://www.openmx-square.org`.
7. Sawahata, H., Yamaguchi, N., Minami, S. & Ishii, F. First-principles calculation of anomalous Hall and Nernst conductivity by local Berry phase. *Phys. Rev. B* **107,** 024404 (2023).
8. Ozaki, T. Variationally optimized atomic orbitals for large-scale electronic structures. *Phys. Rev. B* **67,** 155108 (2003).
9. Mulder, C. A. M., van Duyneveldt, A. J. & Mydosh, J. A. Frequency and field dependence of the ac susceptibility of the AuMn spin-glass. *Phys. Rev. B* **25,** 515–518 (1982).
10. Mydosh, J. A. *Spin Glasses: An Experimental Introduction* (CRC Press, 1993).
11. Anand, V. K., Adroja, D. T. & Hillier, A. D. Ferromagnetic cluster spin-glass behavior in $PrRhSn_3$. *Phys. Rev. B* **85,** 014418 (2012).
12. Bag, P., Baral, P. R. & Nath, R. Cluster spin-glass behavior and memory effect in $Cr_{0.5}Fe_{0.5}Ga$. *Phys. Rev. B* **98,** 144436 (2018).
